# Cariprazine Use in Patients with Neurodevelopmental Disorders Presenting with Obsessive, Ruminative and Stereotyped Behaviors: A Case Series

**DOI:** 10.1192/j.eurpsy.2026.11253

**Published:** 2026-07-31

**Authors:** D. D. Koutsogianni, D. Moschou, V. Bountioukou-Spinari, M. Apostolidis, T. Triantafyllidis, K. Kotsis, P. Petrikis, A. Kοtsi

**Affiliations:** 1 Faculty of Medicine, University Hospital of Ioannina, Ioannina; 2 Psychiatric Clinic, General Hospital of Florina, Florina, Greece

## Abstract

**Introduction:**

Neurodevelopmental disorders (F70–F73: Intellectual disability, F84: Autism spectrum disorders) are often accompanied by obsessive, ruminative, and stereotyped behavioral patterns, which can significantly affect patients’ functioning and quality of life.

Distinguishing between obsessions/ruminations and stereotyped behaviors is often challenging, especially in individuals with limited cognitive or communicative abilities.

Therapeutic options for these symptom dimensions remain limited. Cariprazine, a dopamine D3/D2 and 5-HT1A partial agonist, has shown promising effects on repetitive thoughts, compulsive behaviors, and impulsivity, suggesting potential benefits in patients with neurodevelopmental disorders who present with similar symptom profiles.

**Objectives:**

To describe a series of clinical cases involving patients with neurodevelopmental disorders who presented with repetitive, obsessive, or stereotyped behaviors, and who were treated with cariprazine as either an adjunctive or primary pharmacological intervention. The aim is to explore the clinical response, tolerability, and potential therapeutic role of cariprazine in managing obsessive–ruminative and stereotyped symptomatology within this population.

**Methods:**

**Design:** Case series of patients with ICD-10 diagnoses F70–F72 and F84.

**Inclusion criteria:** Clinically significant repetitive, obsessive, or stereotyped behaviors.

**Intervention:** Cariprazine as adjunctive or monotherapy**,** doses ranging 1.5–4.5 mg/day.

**Follow-up:** 8–12 weeks

**Adverse effects:** Systematically recorded Case 1: male F84 comorbid obsessive symptoms

**Results:**

In this case series, all patients demonstrated meaningful improvement in obsessive, ruminative, or stereotyped behaviors following treatment with cariprazine. Improvements were not limited to symptom reduction, patients also exhibited enhanced mood, increased motivation, greater social engagement, and improved participation in daily activities or structured programs. Notably, cariprazine was well tolerated across the cohort, with no significant adverse effects reported during the follow-up period.

**Discussion:**

The observed clinical response may be attributed to cariprazine’s partial D3/D2 agonist activity, which is hypothesized to modulate dopaminergic circuits implicated in repetitive and compulsive behaviors. These preliminary findings suggest that cariprazine could be a valuable therapeutic option for individuals with neurodevelopmental disorders who exhibit treatment-resistant obsessive or stereotyped symptoms.

**Conclusions:**

Cariprazine appears to be both safe and potentially effective in reducing obsessive and stereotyped behaviors in patients with neurodevelopmental disorders. The improvements in functional engagement, social interaction, and overall quality of life further support its potential utility as part of a comprehensive treatment approach in this challenging patient population.

**Disclosure of Interest:**

None Declared

